# Bacteriostatic effects of benzyl isothiocyanate on *Vibrio parahaemolyticus*: Transcriptomic analysis and morphological verification

**DOI:** 10.1186/s12896-021-00716-4

**Published:** 2021-09-29

**Authors:** Jianan Liu, Ke Zhang, Jie Song, Hongyan Wu, Hongshun Hao, Jingran Bi, Hongman Hou, Gongliang Zhang

**Affiliations:** 1 School of Food Science and Technology, Dalian Polytechnic University, Dalian, 116034 China; 2 Liaoning Key Lab for Aquatic Processing Quality and Safety, Dalian Polytechnic University, Dalian, 116034 China; 3 Department of Inorganic Nonmetallic Materials Engineering, Dalian Polytechnic University, Dalian, 116034 China

**Keywords:** Benzyl isothiocyanate, *Vibrio parahaemolyticus*, RNA-seq, Motility, Biofilm

## Abstract

**Background:**

Foodborne illness caused by *Vibrio parahaemolyticus* (*V. parahaemolyticus*) is generally associated with the consumption of seafood. Fish and other seafood can be contaminated with *V. parahaemolyticus*, natural inhabitants of the marine, estuarine, and freshwater environment. In this study, the antibacterial activities of benzyl isothiocyanate (BITC) against *V. parahaemolyticus* were investigated by both transcriptomic analysis and morphological verification.

**Results:**

Treatment with 1/8 minimum inhibitory concentration (1/8 MIC) BITC resulted in 234 upregulated genes and 273 downregulated genes. The results validated by quantitative real-time polymerase chain reaction (qRT-PCR) revealed that the relative expression levels of the six genes *VP0820, VP0548, VP2233, VPA2362, fliA* and *fliG* were only 31.0%, 31.1%, 55.8%, 57.0%, 75.3%, and 79.9% of the control group, respectively. Among them, genes *VP2233, fliA* and *fliG* are related to flagella and *VP2362* can regulate a protein relevant to biofilm formation. Morphologically, we verified that the swimming diffusion diameter of *V. parahaemolyticus* was significantly reduced by 14.9% by bacterial swimming ability, and biofilm formation was significantly inhibited by treatment with 1/8 MIC BITC by crystal violet quantification assay.

**Conclusions:**

These results indicated that 1/8 MIC BITC had antibacterial effect on *V. parahaemolyticus* by inhibiting virulence gene expression related to flagella and biofilm.

**Supplementary Information:**

The online version contains supplementary material available at 10.1186/s12896-021-00716-4.

## Background

*Vibrio parahaemolyticus* is a moderately halophilic gram-negative bacterium mainly in the form of rods, arcs, etc., without capsules and spores, and has become the main reason of aquatic product poisoning worldwide since it was identified in 1950 [1]. The colonies of *V. parahaemolyticus* CGMCC 1.1614, known as ATCC 33,845, are round, smooth, complete and cream colored. It can grow at temperature of 5 to 44 °C, and survive in the pH range from 4.8 to 11.0. It is common in coastal estuaries and marine environments and is usually associated with seawater and marine organisms such as zooplankton, plankton, mollusks and shellfish, fish and crabs [1]. Bacterial gastroenteritis related to seafood is mainly caused by direct or cross-contamination by *V. parahaemolyticus* [1]. Poisoning usually manifests as diarrhea, headache, vomiting, nausea, abdominal cramps and other symptoms. Therefore, it is necessary to take various measures to reduce the contamination of *V. parahaemolyticus*.

Antibiotics have long been applied to prevent and control marine pollution, but their long-term use has led to bacterial resistance to antibiotics and even the induction of aplastic anemia in human [2]. The hydrolysis of glucosinolate can produce isothiocyanates (ITCs), which are organic sulfides in cruciferous plants. ITCs have shown various beneficial effects, including antibacterial, antiviral, anti-inflammatory, anticancer, neuroprotective, chemical prevention and anti-parasitic properties. Benzyl isothiocyanate (BITC) is a type of ITC strong antibacterial capacity. Studies have found that BITC indicates antifungal activity and can effectively inhibit various molds [3] and bacteria, such as *Campylobacter jejuni* [4], *Salmonella typhimurium* [5], *Staphylococcus aureus* [6]. Studies have indicated the antibacterial mechanism of BITC. For example, BITC can affect the biofilm integrity, bacterial morphology, and membrane potential of *Pseudomonas aeruginosa*, *E. coli* and *S. aureus* [7]. However, knowledge about the antibacterial effects of BITC on *V. parahaemolyticus* is limited.

Scholars have conducted studies on the antibacterial mechanisms of natural products in *V. parahaemolyticus*. The major constituent of black seed volatile oil could affect quorum sensing, biofilm, and virulence-associated genes to reduce the virulence of *V. parahaemolyticus* [8]. Dihydromyricetin is the main bioactive component of Ampelopsis grossedentata, which can inhibit *V. parahaemolyticus* by decreasing the activity of proline dehydrogenase and inducing an increase in cell injury, proline content and cell surface hydrophobicity [9]. Banu et al. [10] studied the effect of essential oil from Cinnamomum tamala on *V. parahaemolyticus* and found that the virulence factors were regulated by polysaccharides, cytotoxins, flagella, lipopolysaccharides and biofilms. Song et al. [11] used the transcriptome to study the antibacterial effect of 1/4 MIC BITC on *V. parahaemolyticus*. However, no study has reported the bacteriostatic effect of BITC on *V. parahaemolyticus* by combining transcriptional level and morphological characteristics. Therefore, the antibacterial mechanism of BITC against *V. parahaemolyticus* can be studied from the genetic level and the destruction of the bacterial membrane.

In the current study, we determined the antibacterial effect of BITC at a 1/8 MIC subinhibitory concentration on *V. parahaemolyticus*. The differentially expressed genes (DEGs) were detected by RNA-seq, from which we screened out genes related to virulence and verified their expression by qRT-PCR. Morphologically, the influence of BITC on *V. parahaemolyticus* motility and biofilm formation was also verified.

## Methods

### Bacterial strain and culture

The *V. parahaemolyticus* CGMCC 1.1614 (*tdh* + , *tlh* + , *trh* -) was purchased from China General Microbiological Culture Collection Center. The strain was stored in physiological saline with 10% glycerol at − 80 °C. 100 µL of frozen bacteria solution was added into 10 mL tryptone soy broth medium with 3% sodium chloride (3% NaCl-TSB) liquid medium and cultured at 37 °C overnight. The cultured bacterial cells were streaked onto tryptone soy agar medium with 3% sodium chloride (3% NaCl-TSA) medium and incubated at 37 °C. Next, the activated single colony was inoculated into 3% NaCl-TSB liquid medium and cultured with shaking at 37 °C.

### Antimicrobial tests

BITC was purchased from Sigma-Aldrich (CAS: 622–78-6), and its Flavor and Extract Manufacturing Association (FEMA) number is 4428. The MIC of BITC of *V. parahaemolyticus* was determined to be 9.54 µmol/L by the broth microdilution method. 1/8 MIC BITC was added to 100 mL 3% NaCl-TSB liquid culture medium with *V. parahaemolyticus* in log phase. Bacterial solution without BITC was incubated at 37 °C on a shaker (150 r/min) for 6 h. Bacterial solution without BITC was used as a control.

### Enrichment and sequencing of RNA

*V. parahaemolyticus* was treated with 1/8 MIC BITC (E_BITC) or without BITC (C_BITC) for 6 h. Total RNA was extracted by the RNAprep Pure Cell/Bacterial Kit (Tiangen Biotech, Beijing, China) as recommended by the manufacturer. The preparation of sequencing library used the Illumina's NEBNext UltraTM Directed RNA Library Preparation Kit (NEB, Ipswich, MA, USA), and then added the index codes. All the samples were evaluated for product and library quality using an Agilent Bioanalyzer 2100 system (G2939B; Agilent Technologies, Palo Alto, CA, USA). The recount data obtained in the gene expression level analysis by DEGseq 2 software were used to analyze and screen the DEGs, wherein the screening standard was *p* < 0.05. DEGs is analyzed using volcanic plots, Gene Ontology (GO) analysis and Kyoto Encyclopedia of Genes and Genomes (KEGG) analysis. The statistical enrichment of DEGs in the KEGG pathway was analyzed by KOBAS software.

### qRT-PCR verification of the RNA-seq results

We used qRT-PCR to investigate the differential gene expression between different groups with *16S rRNA* as the reference gene. The annealing temperature was set at 58 °C and the obtainment of melting curve was 60 to 95 °C. Additionally, the relative expression was calculated by 2^−∆∆Ct^ method. The specific primer sequences for qRT-PCR are listed in Additional file 1. The genomic DNA was removed from the total RNA as recommended by instructions. The cDNA templates were reverse transcribed using the PrimeScript™ RT Reagent kit with gDNA Eraser (TaKaRa, Dalian, China). qRT-PCR was performed by using SYBR®Premix Ex Taq™II (TliRNaseH Plus) (Takara, Dalian, China) as recommended by the manufacturer.

### Mobility measurement

The mobility of *V. parahaemolyticus* was determined based on the method of Butler et al. [12]. One milliliter of the log phase of *V. parahaemolyticus* was added to 99 mL of fresh 3% NaCl-TSB liquid medium, followed by incubation for 6 h. Next, 15 mL of swimming medium was sterilized and cooled to approximately 45 °C, then added 500 μL of 1/8 MIC BITC stock solution or physiological salin as the experimental group or control group, respectively. Thereafter, 3 μL of bacterial solution was added dropwise to the plate, and then the diameter of the inhibition zone was counted after incubation at 37 °C for 12 h. The average diameters of inhibition zone of the control group and experimental group were obtained to show the antibacterial effect of BITC on *V. parahaemolyticus* mobility. The data comparison of each group was analyzed by student’s *t* test. *P* < 0.05 indicated a statistically significant difference.

### Biofilm measurement

The biofilm formation of *V. parahaemolyticus* affected by BITC was investigated according to the method of Pratt and Kolter [13]. Three-percent NaCl-TSB liquid medium, bacterial solution and 1/8 MIC BITC were added to a 96-well plate at ratio of 8:1:1, followed by incubating at 25 °C to form the biofilm. 3% NaCl-TSB liquid medium with and without bacterial culture was used as the controls. After treatment with phosphate buffer saline (PBS), anhydrous methanol, crystal violet and glacial acetic acid, the absorbance was measured at 590 nm using a microplate reader (Spectra Max M2, Molecular Devices, CA, USA). The experiment was repeated three times, and each group included three parallel determinations. The data comparison of two groups was analyzed by student’s *t* test. *P* < 0.05 indicated a statistically significant difference.

## Results

### Transcriptome results

In our previous studies, Song et al. [11] determined the sensitivity of *V. parahaemolyticus* CGMCC 1.1614 to BITC, and the MIC was 9.54 μmol/L. Transcriptome analysis can reveal the underlying molecular mechanisms by which food additives act on pathogens. We performed transcriptome sequencing by the Illumina Hi Seq TM2500 high-throughput sequencing platform, generating two transcriptome databases for the control group without BITC (C_BITC) and the experimental group with 1/8 MIC BITC treatment (E_BITC) to understand the bacteriostatic effect of BITC on *V. parahaemolyticus*. The RNA-seq results were uploaded to the Gene Expression Omnibus (GEO) database, numbered GSE152671. The total quantity of sequencing data we obtained was 9.52 Gb, the error rate of the single-base position of all samples was less than 1%, and Q20 was greater than 80%, which were up to the standards of sequencing quality control (see Additional file 2).

**Fig. 1:**
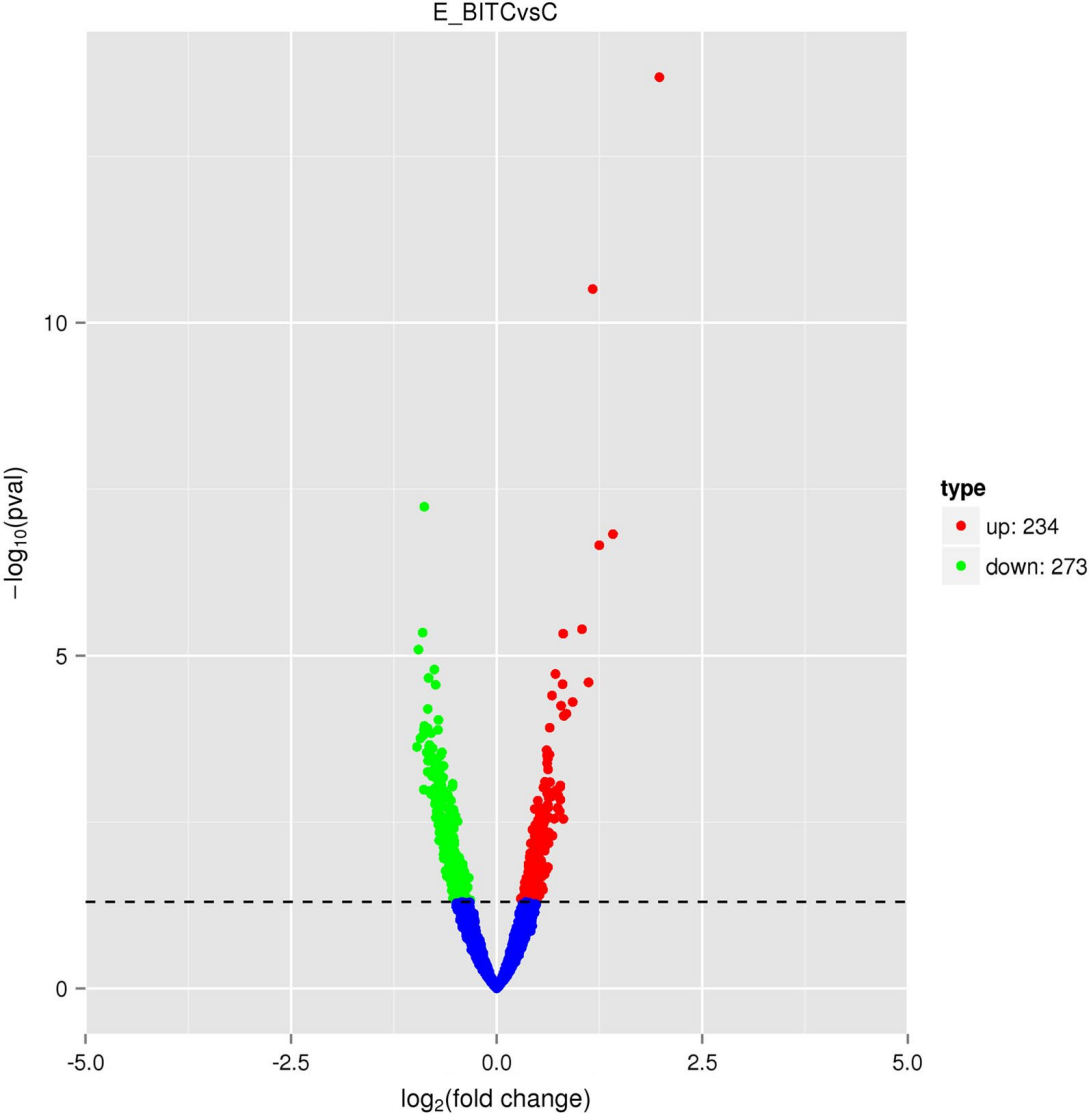
Volcano plot of differentially expressed genes (DEGs) in *Vibrio parahaemolyticus* treated with 1/8 MIC BITC

The volcano map shows the distribution of DEGs. According to Fig. 1, E_BITC obtained 507 DEGs compared with C_BITC, among which 234 were upregulated and 273 were downregulated.

The top 10% genes with the most significantly differential expression were shown in the Table 1, of which 19 were up-regulated and 31 were down-regulated. It was shown that BITC regulated 14 enzyme-related genes, including cryptic beta-D-galactosidase subunit alpha, membrane-bound lytic murein transglycosylase D, orotate phosphoribosyltransferase, long-chain-fatty-acid-CoA ligase, manganese-dependent inorganic pyrophosphatase, oxaloacetate decarboxylase subunit gamma, carbamoyl phosphate synthase small subunit, GTP cyclohydrolase I, ribonuclease PH, quinolinate phosphoribosyltransferase, 5'-deoxynucleotidase, short chain dehydrogenase, glycerol kinase and cytidylate kinase. Oxaloacetate decarboxylase α/β/γ complex is a membrane-bound enzyme complex in *Vibrio cholerae,* and its assembly is affected the oxaloacetate decarboxylase subunit gamma regulated by *VP2545* [14]. The influence of BITC on the activities of these enzyme-related genes should be discussed in the further study. BITC also showed effects on the other genes related to bacterial virulence. For examples, BITC upregulated molecular chaperone DnaK, which is related to protein folding [15]. BITC also affected the expression of transfer protein genes such as *uhpT* and *VP2869*, which regulate sugar phosphate antiporter and sodium/solute symporter, respectively (Table 1). Membrane associated protein genes such as *VP1286* and *VP1091*, which regulate integral membrane protein and transmembrane protein, respectively, affecting cell membrane permeability, biofilm formation and septum formation (Table 1). BITC also regulated some functional proteins, such as sodium/solute symporter, carbon starvation protein A, integral membrane protein, heavy metal membrane efflux protein, RhlE protein, sugar phosphate antiporter, transmembrane protein affecting septum formation and cell membrane permeability, lipoprotein, heat shock protein 90, phosphate ABC transporter ATP-binding protein, integral membrane protein transporter, SpoOM-like protein, 30S ribosomal protein S21, thiamine biosynthesis protein ThiC, NadC family protein and nucleoid occlusion protein. Among them *VP0821*-regulated heat shock protein 90 is important for the virulence and spread of protozoan parasites [16].

**Table 1:** Top 10% genes with significant differential expression identified in E_BITC and C_BITC from RNA sequencing

Gene ID	Gene	Protein function	Log_2_ fold change	*p*-value (10^–3^)	Significant
**Enzyme-related genes**					
VP2403	*ebgA*	Cryptic beta-D-galactosidase subunit alpha	− 0.96863	0.23446	DOWN
VP2296		Membrane-bound lytic murein transglycosylase D	− 0.95036	0.00809	DOWN
VP0178	*pyrE*	Orotate phosphoribosyltransferase	− 0.92727	0.17420	DOWN
VP0351		Long-chain-fatty-acid-CoA ligase	− 0.89877	0.00450	DOWN
VP1165		Manganese-dependent inorganic pyrophosphatase	− 0.89342	0.15740	DOWN
VP2545		Oxaloacetate decarboxylase subunit gamma	− 0.84906	0.28461	DOWN
VP0470		Carbamoyl phosphate synthase small subunit	− 0.83836	0.55676	DOWN
VPA1169	*folE*	GTP cyclohydrolase I	− 0.83411	0.37947	DOWN
VP0177	*rph*	Ribonuclease PH	− 0.81994	0.22615	DOWN
VP2522		Quinolinate phosphoribosyltransferase	− 0.78394	0.64769	DOWN
VP0926		5'-deoxynucleotidase	0.77483	0.89284	UP
VP2120		Short chain dehydrogenase	− 0.75714	0.01611	DOWN
VP2386	*glpK*	Glycerol kinase	0.74715	1.25960	UP
VP2031	*cmk*	Cytidylate kinase	− 0.73197	0.69765	DOWN
**Protein-related genes**					
VP2869		Sodium/solute symporter	1.2476	0.00022	UP
VP0540		Carbon starvation protein A	1.1688	0.31199E-7	UP
VP1286		Integral membrane protein	1.0379	0.00400	UP
VPA0496		Heavy metal membrane efflux protein	0.92515	0.04963	UP
VP0903		RhlE protein	− 0.88603	1.03030	DOWN
VPA0963	*uhpT*	Sugar phosphate antiporter	− 0.88364	0.12876	DOWN
VP1091		Transmembrane protein affecting septum formation and cell membrane permeability	− 0.83853	0.12352	DOWN
VP1267		Lipoprotein	− 0.82293	1.08150	DOWN
VP0821		Heat shock protein 90	0.81662	0.08014	UP
VPA1458		Phosphate ABC transporter ATP-binding protein	0.81113	2.83860	UP
VP3027		Thiamine biosynthesis protein ThiC	− 0.77935	0.24646	DOWN
VPA1704		Integral membrane protein transporter	0.76615	2.18070	UP
VP1278		SpoOM-like protein	− 0.75022	1.68430	DOWN
VP0407	*rpsU*	30S ribosomal protein S21	− 0.75017	0.47875	DOWN
VP1256		NadC family protein	− 0.74166	0.02730	DOWN
VP0180	*slmA*	Nucleoid occlusion protein	− 0.73893	2.70710	DOWN
**Bacterial chemotaxis**					
VP2248	*fliG*	Flagellar motor switch protein G	− 0.79604	0.14531	DOWN
VP1892		Methyl-accepting chemotaxis protein	− 0.82761	0.02157	DOWN
**Hypothetical protein**					
VP1677		Hypothetical protein	1.9793	0.20439E-10	UP
VP2868		Hypothetical protein	1.4143	0.00015	UP
VP1679		Hypothetical protein	1.1181	0.02512	UP
VP1238		Hypothetical protein	− 0.88005	0.58264E-4	DOWN
VPA0208		Hypothetical protein	− 0.87862	0.11346	DOWN
VP0962		Hypothetical protein	0.84754	0.07430	UP
VPA1370		Hypothetical protein	− 0.83752	0.06318	DOWN
VPA0969		Hypothetical protein	0.80972	0.00466	UP
VPA0521		Hypothetical protein	− 0.79721	1.20070	DOWN
VP1380		Hypothetical protein	0.78381	0.05645	UP
VPA0114		Hypothetical protein	0.77526	1.45350	UP
VP1288		Hypothetical protein	0.7717	0.94068	UP
VP1980		Hypothetical protein	− 0.77034	0.34407	DOWN
VPA1613		Hypothetical protein	− 0.7494	1.35230	DOWN
VP1287		Hypothetical protein	0.7427	1.93820	UP
**Other genes**					
VP2826		Transporter	− 0.81597	0.21954	DOWN
VPA1006		LysR family transcriptional regulator	− 0.75038	0.97802	DOWN
VP0653	*dnaK*	Molecular chaperone DnaK	0.80187	0.02678	UP

Besides, rpsU-encoded ribosomal protein S21 can affect the motility and biofilm formation of *Bacillus subtilis* [17]. The down-regulated genes *VP1892* and *FliG* are related to bacterial chemotaxis (see Additional file 3). *FliG* regulates flagellar motor switch protein G (Table 2) and *VP1892* regulates methyl-accepting chemotaxis protein. *VP2826* and *VPA1006* were down-regulated, regulating transporter and LysR family transcriptional regulator, respectively. According to the objective of present study, we focused on the antibacterial mechanism of BITC by screening differentially expressed genes related to virulence, as shown in Table 2 (*VP0820, VP0548, VP2233, VPA2362, fliA* and *fliG*), in which *VP0820*, *VP0548* can regulate ToxR, thereby affecting the virulence of *V. parahaemolyticus*; *VP2233*, *fliA* and *fliG* genes are related to flagella, thereby affecting motility, and *VP2362*-encoded outer membrane protein that is related to the biofilm formation.

**Table 2:** Differentially expressed genes relevant to virulence from RNA sequencing

Gene_ID	Gene name	Log2FoldChange (E_BITC vs. C_BITC)	Pval (E_BITC vs. C_BITC)	Padj (E_BITC vs. C_BITC)	Significant (E_BITC vs. C_BITC)	Description
VP0820	-	− 0.51604	0.022146	0.20191	DOWN	ToxR protein
VP0548	-	− 0.49163	0.027226	0.22101	DOWN	ToxR-activated protein TagE
VP2362	-	− 0.48862	0.020656	0.1966	DOWN	outer membrane protein OmpK
VP2233	-	− 0.44564	0.01859	0.18505	DOWN	flagellar biosynthesis protein FlhG
VP2232	*fliA*	− 0.64333	0.0044437	0.085813	DOWN	flagellar biosynthesis sigma factor
VP2248	*fliG*	− 0.79604	0.00014531	0.015756	DOWN	flagellar motor switch protein G

**Fig. 2:**
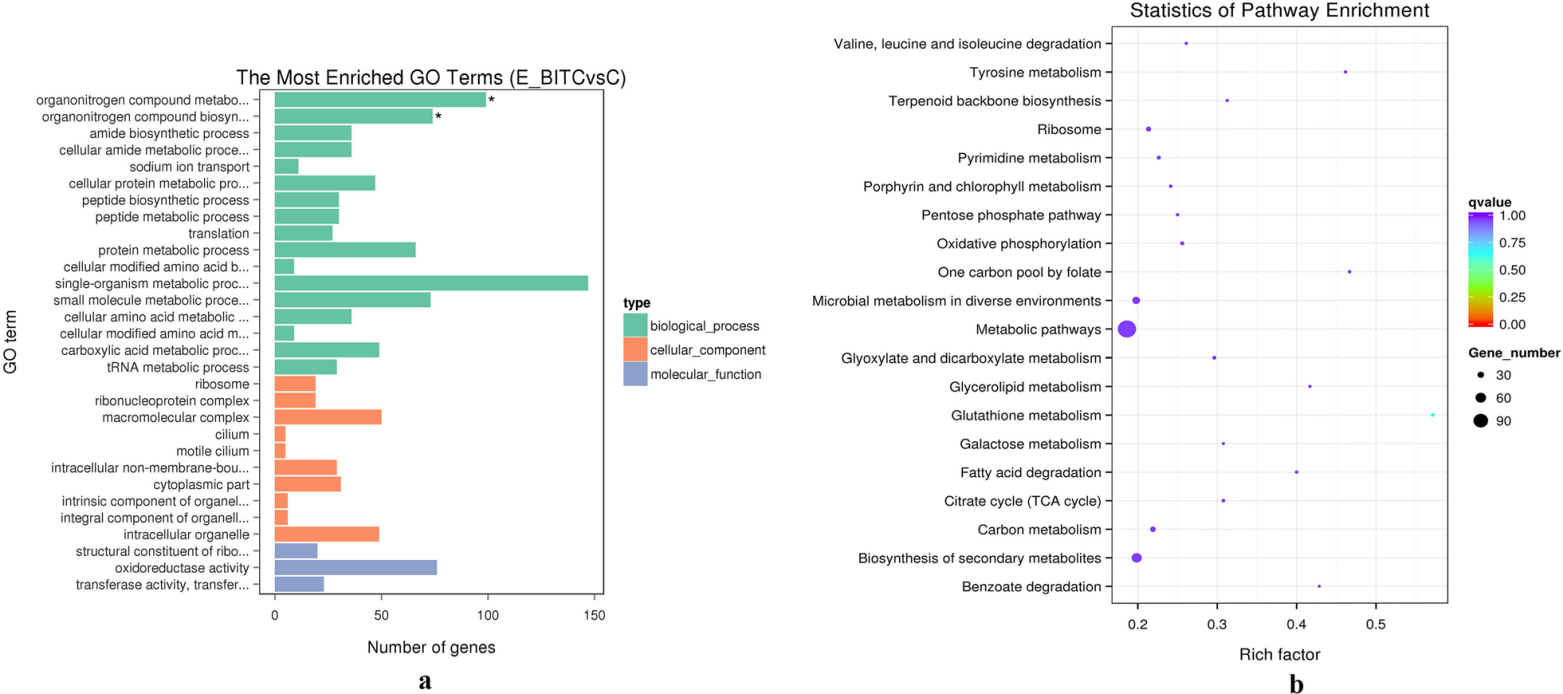
GO analysis (**a**) and KEGG analysis (**b**) of differentially expressed genes (DEGs). **a** Thirty significantly enriched GO terms are shown. ^*^Means significantly enriched GO terms. **b** The 20 special KEGG pathways above include 507 single genes. The closer the *q*-val is to zero, the higher is the degree of gene enrichment

GO analysis was performed on the DEGs in *V. parahaemolyticus* treated with 1/8 MIC BITC. We found that these genes were annotated into 1679 GO terms, and the enrichment was selected from each group. Thirty significant GO terms were identified, with three main domains, molecular functions (12 subclasses), biological processes (10 subclasses) and cellular components (8 subclasses). Among these, 891 were annotated to biological processes, 227 were annotated to cellular components, and 561 were annotated to molecular function. Figure 2a is the most significantly enriched composition selected from each domain. Among them, the macromolecular complex in the cellular component had 23 genes upregulated and 27 genes downregulated. Six upregulated genes *rplC, rplD, rplW, rplB, rpsS* and *rplV*, and 11 downregulated genes *rpsR, rpsU, rpsF, rpsI, rplM, rplN, RplU, VP1210, rpmA, rpmE* and *rpmG* all regulate ribosome-related proteins (see Additional file 4). Studies have shown that ribosomal proteins can affect bacterial motility and biofilm formation [17]. Downregulated genes *VP1892, VP1904, VP2629* and *VPA1000* all regulate the methyl-accepting chemotaxis protein (see Additional file 4), it is related to flagellar movement [18]. Both cilium and motile cilium had significant changes in 5 genes, the upregulated gene *VP1392* and downregulated genes *VP0246*, *VP0417, alaS* and *VP2629* respectively affected the synthesis of cilia-related ClpA/B-type protease, hypothetical protein, hypothetical protein, alanyl-tRNA synthetase, and methyl-accepting chemotaxis protein to influence motility (see Additional file 4). Six genes were significant changed in the groups of intrinsic component of organelle membrane and integral component of organelle membrane. The upregulated gene *metF* regulates 5, 10-methylenetetrahydrofolate reductase, and downregulated genes *VP0388, VP0246, VP0470, VP0939,* and *VPA1370* regulate type I restriction enzyme M protein, hypothetical protein, carbamoyl phosphate synthase small subunit, hypothetical protein and hypothetical protein, respectively (see Additional file 4). Three genes *VP0295, VPA1128,* and *VPA1735* were upregulated, while eight genes *VP1092, VP1256, VP1741, nhaB, VP2351, VP2545, VP2778,* and *VP2826* were downregulated in sodium ion transport of biological processes. They regulated multiple proteins related to sodium ion transport, such as sodium/sulfate symporter, acyl-CoA carboxylase alpha chain, acridine efflux pump, NadC family protein, sodium/alanine symporter, sodium/proton antiporter, Na( +)-translocating NADH-quinone reductase subunit A, oxaloacetate decarboxylase subunit gamma, FKBP-type peptidylprolyl isomerase and transporter (see Additional file 4). In the molecular function, oxidoreductase activity had 32 upregulated genes and 44 downregulated genes, transferase activity, transferring one-carbon groups had 4 upregulated genes and 19 downregulated genes. Multiple genes related to oxidoreductase such as *VP0068, VP0235, VP1017, VPA0278, phhA, VP0442, sdhC, VP1710, VP2014,* and *VPA0566*, which regulate glutathione reductase, epimerase/dehydratase, arginyl-tRNA-protein transferase, isopentenyl pyrophosphate isomerase, phenylalanine 4-monooxygenase, ubiquinol-cytochrome c reductase, cytochrome b, succinate dehydrogenase cytochrome b556 large membrane subunit, glucose-6-phosphate 1-dehydrogenase, tetrathionate reductase subunit A and alcohol dehydrogenase, respectively (see Additional file 4). Among the DEGs in the term of transferase activity, transferring one-carbon groups, there are multiple methyltransferase-related genes such as *VPA0046, ubiE, VP0594, VP0954, yebU, VP1933, VP2477,* and *gidB*, which regulate methylated-DNA–protein -cysteine S-methyltransferase, ubiquinone/menaquinone biosynthesis methyltransferase, RNA methyltransferase, *16S rRNA* (cytosine(1407)-C(5))-methyltransferase RsmF, 3-demethylubiquinone-9 3-methyltransferase, *16S rRNA* methyltransferase and *16S rRNA* methyltransferase GidB (see Additional file 4). Some studies have confirmed that methyltransferase can affect bacterial motility and biofilm formation [19]. These terms might be related to the motility and biofilm formation in *V. parahaemolyticus*.

The analysis of KEGG enrichment was conducted following GO analysis. In total, 507 DEGs were enriched into 78 pathways compared with the control group. Bacterial chemotaxis pathways can affect the direction of bacterial movement by adjusting the rotation direction of flagella movement. Bacterial chemotaxis pathways included 7 differentially expressed genes, of which *VP1628, VP1892, VP1904, VP2629,* and *VPA1000* regulate methyl-accepting chemotaxis protein, while *VP2230* and *fliG* regulate chemotaxis protein CheZ and flagellar motor switch protein G, respectively (see Additional file 3). Flagellar assembly pathways included three DEGs *flgF, fliI,* and *fliG*, which are related to flagellar basal body rod protein FlgF, flagellar-specific ATP synthase and flagellar motor switch protein G, respectively (see Additional file 3). Figure 2b shows 20 pathways with the most enrichment, such as ABC transporters, biosynthesis of secondary metabolites, carbon metabolism, microbial metabolism in diverse environments and metabolic pathways.

### qRT-PCR verification of the RNA-seq results

**Fig. 3:**
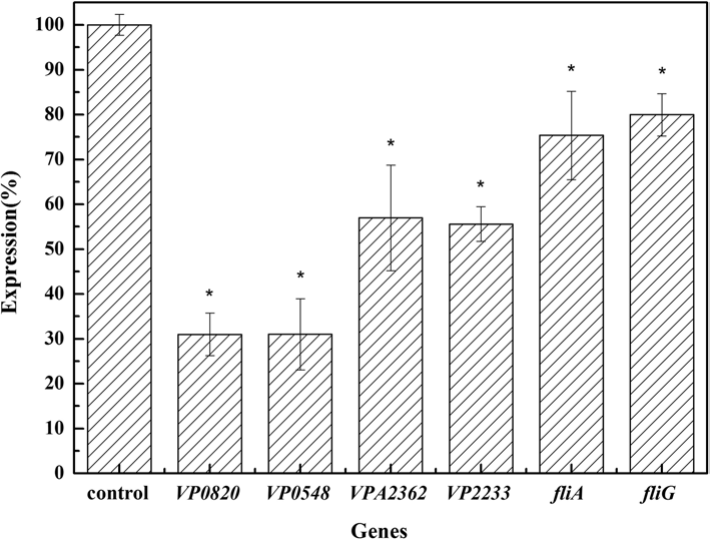
Inhibition of the gene expressions related to virulrnce in *V. parahaemolyticus* by 1/8 MIC BITC. *VP0820* and *VP0548* encode the ToxR protein gene, *VP2233, fliA* and *fliG* are related genes of flagellin synthesis, and *VPA2362* encodes the outer membrane protein gene. The data in the figure were derived from the average of three parallel experiments. *p < 0.05 indicates significant differences

qRT-PCR is a common and important technical method to study the gene expression levels. The DEGs related to virulence were screened by RNA-seq, and the reliability of the data was verified by qRT-PCR. According to RNA-seq, six genes related to virulence with obviously decreased expression were screened (Table 2). The expression of six virulence-related genes was investigated in *V. parahaemolyticus* treated with 1/8 MIC BITC by qRT-PCR. The virulence-related genes *VP0820, VP0548, VP2233, VPA2362, fliA,* and *fliG* were downregulated (Fig. 3). The relative expression of these genes was significantly reduced with values of 31.0%, 31.1%, 55.8%, 57.0%, 75.3%, and 79.9% of the control group, respectively. These results verified that BITC at 1/8 MIC could effectively inhibit virulence-related gene expression of *V. parahaemolyticus*.

### Mobility measurement

**Fig. 4:**
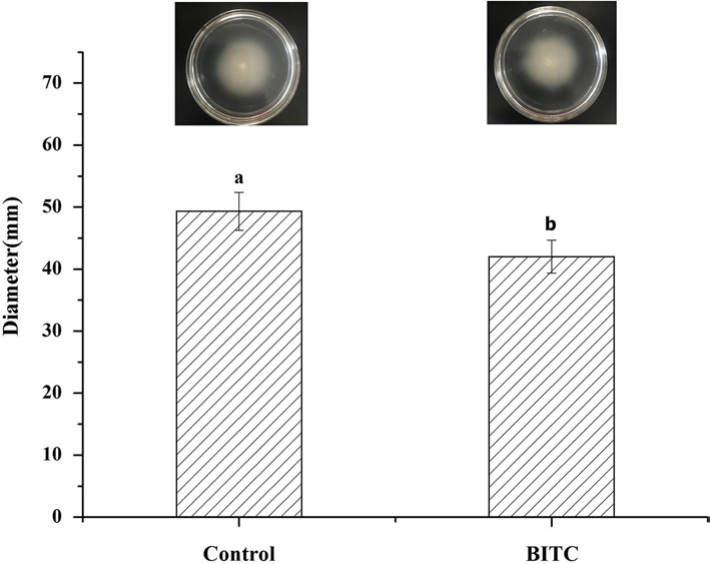
Inhibitory influences of BITC on the swimming ability of *V. parahaemolyticus*. The data in the figure were derived from the average of three parallel

The motility of *V. parahaemolyticus* is affected by the flagella of bacterium, which are connected with the virulence of bacteria. According to the transcriptome results, the expression of flagella-related genes *VP2233, fliA,* and *fliG* was downregulated. By detecting the movement of *V. parahaemolyticus*, we verified the antibacterial effect of BITC on *V. parahaemolyticus* morphologically. The motility of *V. parahaemolyticus* is related to the flagella of bacterium. As shown in Fig. 4, BITC at 1/8 MIC significantly suppressed the mobility of *V. parahaemolyticus*. BITC significantly reduced the diameter of swimming diffusion by 14.9% (*p* < 0.05) compared with that of control group. This finding agreed with the downregulation of *VP2233, fliA,* and *fliG* gene expression in the transcriptome results (Table 2). Therefore, swimming is related to the bacteriostatic effect of BITC in *V. parahaemolyticus*.

### Biofilm measurement

**Fig. 5:**
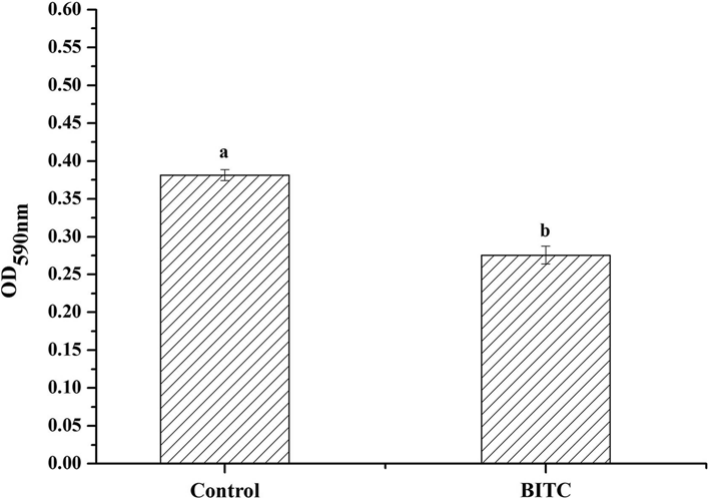
Inhibitory influences of BITC on the biofilm formation of *V. parahaemolyticus*. The data in the figure were derived from the average of three parallel

The formation of biofilm is related to the virulence of bacteria. In most cases, bacteria adhere to the surface of object during formation of biofilms to maintain survival, causing food safety problems. The mechanism of BITC in food-borne pathogenic bacteria can be investigated by exploring the biofilm changes of *V. parahaemolyticus* after BITC treatment. *VP2362* encodes an outer membrane protein, which is related to the biofilm formation of *V. parahaemolyticus*. According to the transcriptome results, the relative expression of this gene was downregulated by treatment with BITC. We expected to verify the transcriptome results by measuring the biofilm formation of *V. parahaemolyticus* and understand the mechanism of BITC effects. The formation of biofilm of *V. parahaemolyticus* was significantly inhibited by 1/8 MIC BITC treatment (*p* < 0.05), and BITC functioned to scavenge biofilm (Fig. 5). These results were consistent with the downregulation of *VP2362* gene expression (see Additional file 4).

## Discussion

Transcriptome sequencing technology is an important technical approach to screen DEGs and determine gene functions. It has advantages in terms of time, data yield, cost, coverage, and accuracy of data [20]. It is also possible to obtain gene-related biological function data by analyzing multilevel gene regulation expression. Studies based on prokaryotic transcriptome sequencing have included *E. coli* [21], *S. aureus* [22], and *Listeria monocytogenes* [23]. Moreover, the difference in gene expression of *V. parahaemolyticus* in seawater has been studied under different oxygen environments [24]. Therefore, RNA sequencing was applied in this study to understand the corresponding changes of *V. parahaemolyticus* CGMCC 1.1614 under the action of 1/8 MIC BITC. According to the research purpose, we screened the DEGs related to virulence at the molecular level and then verified the gene expression using qRT-PCR. From a molecular biology perspective, the impact of BITC on both the virulence and morphology of *V. parahaemolyticus* was also investigated. Studies have shown that the motility of bacteria will be inhibited when sodium ion transport is disturbed [25]. Oxidoreductase and transferase are very important in the formation of biofilms [26, 27]. RNA sequencing results of *Salmonella Enteritidis* under acid stress have shown that the terms of macromolecular complexes, transferase activity, and transferring one-carbon groups are the most highly represented [28]. Combined with the GO analysis results, multiple pathways related to motility and biofilm were significantly enriched (sodium ion transport, oxidoreductase activity, transferase activity, transferring one-carbon groups and macromolecular complex). Therefore, it is necessary to investigate the influence of BITC on *V. parahaemolyticus* of motility and biofilm formation.

Recent findings have confirmed that flagella are adhesive and invasive as a potential class of virulence factors [29]. Bacterial motility is closely related to flagella, and *V. parahaemolyticus* has two different types of flagella to accommodate life in different situations [24]. Studies have shown that bacterial motility is also inhibited when flagella are affected. When histone-like nucleoid structuring protein (H-NS) acted on the flagellin *lateral flagellar A (lafA)*, the swarming motilities of *Vibrio hemolyticus* [30] was inhibited. Quinazoline-2, 4-diamino analogs inhibit the aggregation of polar flagella and movement of *V. cholerae* [31], and phenethyl isothiocyanate (PEITC) can reduce the migration capacity of *E. coli* [32]. In this study, the transcriptome results indicated that the flagella-related gene expression of *VP2233, VP2232,* and *VP2248* were significantly inhibited by 1/8 MIC BITC treatment. Additionally, the morphological verification results of Fig. 4 were similar to the transcriptome results, further proving that the flagella and bacterial motility are related to the bacteriostatic effects of BITC on *V. parahaemolyticus*.

The antimicrobial effects of ITCs are thought to be associated with damage to bacterial membrane integrity [33]. ITCs, including BITC, prevent the formation of biofilms in various bacteria such as *P. aeruginosa, L. monocytogenes, S. aureus,* and *E. coli* [32]. *VP2362* is a gene encoding an outer membrane protein, which is connected with the biofilm formation of *V. parahaemolyticus*. By BITC treatment, gene expression level of *VP2362* was significantly downregulated compared with that of control group. Previous findings have confirmed that the cell surface integrity, neutralizing host defense mechanisms, cell adhesion and invasion, and inhibition of the complement system are all affected by outer membrane proteins [34]. Therefore, the outer membrane protein is considered to be a virulence factor. The expression of membrane genes was significantly downregulated after BITC treatment at 1/8 MIC (*p* < 0.05). We found that the formation of *V. parahaemolyticus* of biofilm was inhibited by BITC treatment, which was consistent with the gene expression results.

In this paper, the bacteriostatic effect of BITC on *V. parahaemolyticus* was reported by RNA-seq technology from the gene level, showing multiple pathways of secondary metabolites. Various virulence genes were inhibited that were further verified morphologically. Further studies will focus on other important DEGs and the corresponding proteomics studies to obtain more comprehensive mechanism for bacteriostatic effects of BITC.

## Conclusions

In this paper, the bacteriostatic effect of BITC on *V. parahaemolyticus* was reported by RNA-seq technology from the gene level, showing multiple pathways of secondary metabolites. Various virulence genes were inhibited that were further verified morphologically. Further studies will focus on other important DEGs and the corresponding proteomics studies to obtain more comprehensive mechanism for bacteriostatic effects of BITC.

## Supplementary Information

**Additional file 1.** Sequences of specific primers for qRT–PCR (doc).

**Additional file 2.** Data of RNA sequencing (doc).

**Additional file 3.** Differentially expressed genes related to bacterial motility from KEGG pathways (doc).

**Additional file 4.** Differentially expressed genes from GO terms (doc).

## Data Availability

All data generated or analyzed during this study are included in this published article.

## Abbreviations

BITC: Benzyl isothiocyanate
E_BITC: Experimental group
C_BITC: Control group
MIC: Minimum inhibitory concentration
qRT-PCR: Quantitative real-time PCR
ITCs: Isothiocyanates
DEGs: Differentially expressed genes
GO: Gene ontology
KEGG: Kyoto Encyclopedia of Genes and Genomes
PBS: Phosphate buffer saline
GEO: Gene expression omnibus
