# Supplementary material for: Bacteriostatic effects of benzyl isothiocyanate on Vibrio parahaemolyticus: Transcriptomic analysis and morphological verification
Source: BMC Biotechnol. 2021 Sep 29;21:56. doi: 10.1186/s12896-021-00716-4 (PMC8479925; doi:10.1186/s12896-021-00716-4)
Supplement: Supplementary file 1 — Additional file 1. Sequences of specific primers for qRT–PCR (doc). [file 12896_2021_716_MOESM1_ESM.docx]

**Additional file 1** Sequences of specific primers for qRT–PCR

| **Gene** | **Primer** | **Sequence (5’-3’)** |
| --- | --- | --- |
| *16S rRNA* | 16S rRNA-F | TATCCTTGTTTGCCAGCGAG |
|  | 16S rRNA-R | CTACGACGCACTTTTTGGGA |
| *VP0820* | VP0820-F | ATTCGCTCGCTGACCAACAAAG |
|  | VP0820-R | ACGCCAAACAAACTCGTGAAGC |
| *VP0548* | VP0548-F | AGCGAGAAAGCAGAAATCACAG |
|  | VP0548-R | ACCGATTCCACATCAAATACGC |
| *VP2233* | VP2233-F | CGCATTTGGTAGTCTTGAA |
|  | VP2233-R | AGAAGTCGGTTCGTCACAG |
| *VP2362* | VP2362-F | TCAGACAAAGGCAGCGACAA |
|  | VP2362-R | ACCATCCCACTCAAATAGCG |
| *fliA* | fliA-F | CGTATTGCTCACCACCTGTTA |
|  | fliA-R | CTCGCACCTTTAGAACCATCA |
