# Supplementary material for: Bacteriostatic effects of benzyl isothiocyanate on Vibrio parahaemolyticus: Transcriptomic analysis and morphological verification
Source: BMC Biotechnol. 2021 Sep 29;21:56. doi: 10.1186/s12896-021-00716-4 (PMC8479925; doi:10.1186/s12896-021-00716-4)
Supplement: Supplementary file 2 — Additional file 2. Data of RNA sequencing (doc). [file 12896_2021_716_MOESM2_ESM.docx]

**Additional file 2.** Data of RNA sequencing

| **Sample name^*^** | **Raw reads** | **Clean reads** | **Clean bases** | **Error rate (%)** | **Q20**  **(%)** | **Q30**  **(%)** | **GC content (%)** |
| --- | --- | --- | --- | --- | --- | --- | --- |
| C_BITC1 | 11409316 | 10480246 | 1.57G | 0.02 | 97.13 | 92.51 | 48.29 |
| C_BITC2 | 12152830 | 11176478 | 1.68G | 0.02 | 96.93 | 92.11 | 48.39 |
| C_BITC3 | 11803196 | 11105878 | 1.67G | 0.02 | 97.00 | 92.28 | 48.35 |
| E_BITC1 | 12200314 | 11242146 | 1.69G | 0.02 | 96.92 | 92.09 | 48.23 |
| E_BITC2 | 10891634 | 10076990 | 1.51G | 0.02 | 97.08 | 92.44 | 47.81 |
| E_BITC3 | 10165134 | 9302252 | 1.40G | 0.02 | 97.08 | 92.43 | 48.37 |

Q20, Q30 respectively represent the percentage of bases with a quality value ≧ 20 or 30. C_BITC: control group without BITC; E_BITC: experimental group with 1/8 MIC BITC treatment. ^*^The control group is the same as shown in Song et al. (2019)
