# Supplementary material for: Bacteriostatic effects of benzyl isothiocyanate on Vibrio parahaemolyticus: Transcriptomic analysis and morphological verification
Source: BMC Biotechnol. 2021 Sep 29;21:56. doi: 10.1186/s12896-021-00716-4 (PMC8479925; doi:10.1186/s12896-021-00716-4)
Supplement: Supplementary file 3 — Additional file 3. Differentially expressed genes related to bacterial motility from KEGG pathways (doc). [file 12896_2021_716_MOESM3_ESM.docx]

**Additional file 3** Differentially expressed genes related to bacterial motility from KEGG pathways

| **Gene ID** | **Gene** | **Protein Function** | **Log2 fold change** | ***p*-value** | **Significant** |
| --- | --- | --- | --- | --- | --- |
| **Bacterial chemotaxis** | | | | | |
| VP1628 |  | methyl-accepting chemotaxis protein | -0.4921 | 0.018704 | DOWN |
| VP1892 |  | methyl-accepting chemotaxis protein | -0.82761 | 2.16E-05 | DOWN |
| VP1904 |  | methyl-accepting chemotaxis protein | -0.56608 | 0.0082365 | DOWN |
| VP2230 |  | chemotaxis protein CheZ | -0.43187 | 0.048811 | DOWN |
| VP2248 | *fliG* | flagellar motor switch protein G | -0.79604 | 0.00014531 | DOWN |
| VP2629 |  | methyl-accepting chemotaxis protein | -0.566 | 0.020225 | DOWN |
| VPA1000 |  | methyl-accepting chemotaxis protein | -0.45491 | 0.023071 | DOWN |
| **Flagellar assembly** | | | | | |
| VP0780 | *flgF* | flagellar basal body rod protein FlgF | 0.82525 | 0.0029964 | UP |
| VP2246 | *fliI* | flagellum-specific ATP synthase | -0.49453 | 0.021258 | DOWN |
| VP2248 | *fliG* | flagellar motor switch protein G | -0.79604 | 0.00014531 | DOWN |
