# Supplementary material for: Bacteriostatic effects of benzyl isothiocyanate on Vibrio parahaemolyticus: Transcriptomic analysis and morphological verification
Source: BMC Biotechnol. 2021 Sep 29;21:56. doi: 10.1186/s12896-021-00716-4 (PMC8479925; doi:10.1186/s12896-021-00716-4)
Supplement: Supplementary file 4 — Additional file 4. Differentially expressed genes from GO terms (doc). [file 12896_2021_716_MOESM4_ESM.docx]

**Additional file 4** Differentially expressed genes from GO terms

| **Gene ID** | ***Gene*** | **Protein Function** | **Log2 fold change** | ***p*-value** | **Significant** |
| --- | --- | --- | --- | --- | --- |
| **Ribosomal protein** | | | | | |
| VP0261 | *rpsS* | 30S ribosomal protein S19 | 0.45286 | 0.021412 | UP |
| VP0260 | *rplB* | 50S ribosomal protein L2 | 0.51376 | 0.0074624 | UP |
| VP0262 | *rplV* | 50S ribosomal protein L22 | 0.49417 | 0.0054398 | UP |
| VP0259 | *rplW* | 50S ribosomal protein L23 | 0.5657 | 0.0027872 | UP |
| VP0257 | *rplC* | 50S ribosomal protein L3 | 0.59403 | 0.0019439 | UP |
| VP0258 | *rplD* | 50S ribosomal protein L4 | 0.62203 | 0.0017299 | UP |
| VP2738 | *rpsR* | 30S ribosomal protein S18 | -0.71655 | 0.00068673 | DOWN |
| VP0407 | *rpsU* | 30S ribosomal protein S21 | -0.75017 | 0.00047875 | DOWN |
| VP2740 | *rpsF* | 30S ribosomal protein S6 | -0.55138 | 0.0088272 | DOWN |
| VP0439 | *rpsI* | 30S ribosomal protein S9 | -0.55858 | 0.01746 | DOWN |
| VP0438 | *rplM* | 50S ribosomal protein L13 | -0.69048 | 0.00058149 | DOWN |
| VP0267 | *rplN* | 50S ribosomal protein L14 | -0.40323 | 0.044358 | DOWN |
| VP0328 | *rplU* | 50S ribosomal protein L21 | -0.48925 | 0.0098943 | DOWN |
| VP1210 |  | 50S ribosomal protein L25 | -0.52641 | 0.010105 | DOWN |
| VP0329 | *rpmA* | 50S ribosomal protein L27 | -0.42921 | 0.028922 | DOWN |
| VP0255 | *rpmE* | 50S ribosomal protein L31 | -0.56221 | 0.011845 | DOWN |
| VP0186 | *rpmG* | 50S ribosomal protein L33 | -0.62546 | 0.0061948 | DOWN |
| **Methyl-accepting chemotaxis protein** | | | | | |
| VP1892 |  | methyl-accepting chemotaxis protein | -0.82761 | 2.16E-05 | DOWN |
| VP1904 |  | methyl-accepting chemotaxis protein | -0.56608 | 0.0082365 | DOWN |
| VP2629 |  | methyl-accepting chemotaxis protein | -0.566 | 0.020225 | DOWN |
| VPA1000 |  | methyl-accepting chemotaxis protein | -0.45491 | 0.023071 | DOWN |
| **Cilium and motile cilium** | | | | | |
| VP1392 |  | ClpA/B-type protease | 0.56476 | 0.0081802 | UP |
| VP0246 |  | hypothetical protein | -0.40492 | 0.047641 | DOWN |
| VP0417 |  | hypothetical protein | -0.70681 | 0.0019519 | DOWN |
| VP2548 | *alaS* | alanyl-tRNA synthetase | -0.49301 | 0.023287 | DOWN |
| VP2629 |  | methyl-accepting chemotaxis protein | -0.566 | 0.020225 | DOWN |
| **Intrinsic component of organelle membrane and integral component of organelle membrane** | | | | | |
| VP2763 | *metF* | 5, 10-methylenetetrahydrofolate reductase | 0.39333 | 0.035315 | UP |
| VP0246 |  | hypothetical protein | -0.40492 | 0.047641 | DOWN |
| VP0388 |  | type I restriction enzyme M protein | -0.69729 | 0.0025797 | DOWN |
| VP0470 |  | carbamoyl phosphate synthase small subunit | -0.83836 | 0.00055676 | DOWN |
| VP0939 |  | hypothetical protein | -0.5932 | 0.0051345 | DOWN |
| VPA1370 |  | hypothetical protein | -0.83752 | 6.32E-05 | DOWN |
| **Sodium ion transport** | | | | | |
| VP0295 |  | sodium/sulfate symporter | 0.39252 | 0.040711 | UP |
| VPA1128 |  | acyl-CoA carboxylase alpha chain | 0.44921 | 0.025016 | UP |
| VPA1735 |  | hypothetical protein | 0.38486 | 0.023923 | UP |
| VP1092 |  | acridine efflux pump | -0.449 | 0.026968 | DOWN |
| VP1256 |  | NadC family protein | -0.74166 | 2.73E-05 | DOWN |
| VP1741 |  | sodium/alanine symporter | -0.44302 | 0.039612 | DOWN |
| VP2072 | *nhaB* | sodium/proton antiporter | -0.64836 | 0.0010515 | DOWN |
| VP2351 |  | Na(+)-translocating NADH-quinone reductase subunit A | -0.53467 | 0.00083996 | DOWN |
| VP2545 |  | oxaloacetate decarboxylase subunit gamma | -0.84906 | 0.00028461 | DOWN |
| VP2778 |  | FKBP-type peptidylprolyl isomerase | -0.44286 | 0.01362 | DOWN |
| VP2826 |  | transporter | -0.81597 | 0.00021954 | DOWN |
| **Oxidoreductase activity** | | | | | |
| VP0068 |  | glutathione reductase | 0.33484 | 0.0316 | UP |
| VP0235 |  | epimerase/dehydratase | 0.33344 | 0.046211 | UP |
| VP1017 |  | arginyl-tRNA-protein transferase | 0.51938 | 0.0071806 | UP |
| VPA0278 |  | isopentenyl pyrophosphate isomerase | 0.42474 | 0.016598 | UP |
| VPA0576 | *phhA* | phenylalanine 4-monooxygenase | 0.54791 | 0.029868 | UP |
| VP0442 |  | ubiquinol-cytochrome c reductase%2C cytochrome b | -0.48454 | 0.025786 | DOWN |
| VP0843 | *sdhC* | succinate dehydrogenase cytochrome b556 large membrane subunit | -0.6464 | 0.0004536 | DOWN |
| VP1710 |  | glucose-6-phosphate 1-dehydrogenase | -0.59373 | 0.01043 | DOWN |
| VP2014 |  | tetrathionate reductase subunit A | -0.40427 | 0.02504 | DOWN |
| VPA0566 |  | alcohol dehydrogenase | -0.70196 | 0.003034 | DOWN |
| **Transferase activity, transferring one-carbon groups** | | | | | |
| VPA0046 |  | methylated-DNA-protein-cysteine S-methyltransferase | 0.45718 | 0.026181 | UP |
| VP0095 | *ubiE* | ubiquinone/menaquinone biosynthesis methyltransferase | -0.61138 | 0.0030322 | DOWN |
| VP0594 |  | RNA methyltransferase | -0.47662 | 0.040601 | DOWN |
| VP0954 |  | RNA methyltransferase | -0.6111 | 0.0079916 | DOWN |
| VP1612 | *yebU* | 16S rRNA (cytosine(1407)-C(5))-methyltransferase RsmF | -0.72949 | 0.0021888 | DOWN |
| VP1933 |  | 3-demethylubiquinone-9 3-methyltransferase | -0.7055 | 0.0016898 | DOWN |
| VP2477 |  | 16S rRNA methyltransferase | -0.55729 | 0.015585 | DOWN |
| VP3079 | *gidB* | 16S rRNA methyltransferase GidB | -0.53276 | 0.0088618 | DOWN |
